# The deubiquitinase USP7 uses a distinct ubiquitin-like domain to deubiquitinate NF-ĸB subunits

**DOI:** 10.1074/jbc.RA120.014113

**Published:** 2020-06-25

**Authors:** Izaskun Mitxitorena, Domenico Somma, Jennifer P. Mitchell, Matti Lepistö, Christian Tyrchan, Emma L. Smith, Patrick A. Kiely, Helen Walden, Karen Keeshan, Ruaidhrí J. Carmody

**Affiliations:** 1 GLAZgo Discovery Centre, Institute of Infection, Immunity & Inflammation, College of Medicine, Veterinary and Life Sciences, University of Glasgow, Glasgow, United Kingdom; 2 Centre for Immunobiology, Institute of Infection, Immunity & Inflammation, College of Medicine, Veterinary and Life Sciences, University of Glasgow, Glasgow, United Kingdom; 3 Rheumatoid Arthritis Pathogenesis Centre of Excellence, Centre for Immunobiology, Institute of Infection, Immunity & Inflammation, University of Glasgow, Glasgow, United Kingdom; 4 Innovative Medicines and Early Development, Respiratory, Inflammation and Autoimmunity, AstraZeneca AB, Gothenburg, Sweden; 5 Graduate Entry Medical School, Health Research Institute and Bernal Institute, University of Limerick, Limerick, Ireland; 6 Institute of Molecular Cell and Systems Biology, College of Medical, Veterinary and Life Sciences, University of Glasgow, Glasgow, United Kingdom; 7 Paul O'Gorman Leukemia Research Centre, Institute of Cancer Sciences, College of Medical, Veterinary and Life Sciences, University of Glasgow, Glasgow, United Kingdom

**Keywords:** ubiquitylation (ubiquitination), ubiquitin-specific peptidase 7 (USP7), NF-kappa B (NF-ĸB), protein–protein interaction, ubiquitin-like domain, deubiquitinase, transcription factor, Toll-like receptor, inflammation, gene regulation, innate immunity, deubiquitylation (deubiquitination)

## Abstract

The transcription factor NF-ĸB is a master regulator of the innate immune response and plays a central role in inflammatory diseases by mediating the expression of pro-inflammatory cytokines. Ubiquitination-triggered proteasomal degradation of DNA-bound NF-ĸB strongly limits the expression of its target genes. Conversely, USP7 (deubiquitinase ubiquitin-specific peptidase 7) opposes the activities of E3 ligases, stabilizes DNA-bound NF-ĸB, and thereby promotes NF-ĸB–mediated transcription. Using gene expression and synthetic peptide arrays on membrane support and overlay analyses, we found here that inhibiting USP7 increases NF-ĸB ubiquitination and degradation, prevents Toll-like receptor–induced pro-inflammatory cytokine expression, and represents an effective strategy for controlling inflammation. However, the broad regulatory roles of USP7 in cell death pathways, chromatin, and DNA damage responses limit the use of catalytic inhibitors of USP7 as anti-inflammatory agents. To this end, we identified an NF-ĸB–binding site in USP7, ubiquitin-like domain 2, that selectively mediates interactions of USP7 with NF-ĸB subunits but is dispensable for interactions with other proteins. Moreover, we found that the amino acids ^757^LDEL^760^ in USP7 critically contribute to the interaction with the p65 subunit of NF-ĸB. Our findings support the notion that USP7 activity could be potentially targeted in a substrate-selective manner through the development of noncatalytic inhibitors of this deubiquitinase to abrogate NF-ĸB activity.

The transcription factor NF-ĸB is a master regulator of inflammation and is essential for the development and homeostasis of the immune system. It is activated by most immunoreceptors including Toll-like receptors (TLRs), antigen receptors and members of the tumor necrosis factor receptor superfamily (1). As a consequence, NF-ĸB is critically important for the transcriptional response to infection and the development of immunity, and at least 500 genes are direct transcriptional targets of NF-κB including cytokines, chemokines, regulators of antigen presentation and cell adhesion, as well as genes that control cell survival, proliferation, and differentiation (1). The functional nature of these transcriptional targets makes NF-κB a central factor in the pathology of a number of important human diseases including chronic inflammatory disease (*e.g.* arthritis, autoimmunity), atherosclerosis, cancer, and neurodegeneration (1). In many of these diseases NF-ĸB is inappropriately activated or active at elevated levels, establishing it as a factor with significant therapeutic potential.

NF-ĸB is in fact a family of transcription factors formed by the dimerization of five subunits; RelA (p65), c-Rel, RelB, p50, and p52. In resting cells, NF-κB is sequestered in the cytoplasm through interaction with the inhibitor of NF-κB (IκB) proteins, of which IκBα is the archetypal member. Activation of the IĸB kinase (IKK) complex (which contains the IKKα and IKKβ kinases and the scaffold protein NEMO) leads to IκBα degradation and the nuclear translocation of NF-κB. Once in the nucleus, NF-κB binds to specific DNA sequences (ĸB sites) to promote transcription (1). The primary mechanism for terminating NF-κB activity is a negative feedback loop involving NF-κB–directed resynthesis of IκBα, which relocates NF-κB from the nucleus to the cytoplasm. There is also a critical IκBα-independent mechanism to limit NF-κB activity that requires the ubiquitination and proteasomal degradation of NF-κB itself (2–4). Ubiquitination of NF-κB occurs in the nucleus and depends on the binding of NF-κB to DNA (2, 5). NF-κB ubiquitination is predominantly composed of K48-linked polyubiquitin chains that trigger proteasomal mediated degradation, leading to reduced NF-κB promoter occupancy and inhibition of transcription (4). The ubiquitination of NF-ĸB with nondegradative ubiquitin linkages has also been described (6); however, at present the functional consequences, if any, are unknown. At least six different E3 ligases for NF-κB have been identified that appear to regulate NF-κB transcriptional activity in a gene selective manner (4, 7–12). The apparent nonredundant roles for these E3 ligases in the regulation of NF-ĸB suggest a complex mechanism for the control of NF-ĸB transcriptional activity by ubiquitination, which remains largely undefined.

The importance of ubiquitination in regulating NF-ĸB transcriptional activity was fully recognized following the identification of USP7 (ubiquitin-specific protease 7) as a deubiquitinase of NF-κB (13). USP7 directly counteracts the activity of E3 ligases by removing polyubiquitin chains from NF-κB, thereby stabilizing NF-ĸB and promoting the transcription of target genes (13). Blocking USP7-mediated deubiquitination of NF-ĸB inhibits NF-ĸB transcriptional activity while inhibiting the E3 ligases of NF-ĸB or the proteasome leads to increased transcription of NF-ĸB target genes (4). Thus, the transcriptional activity of NF-κB is determined by a balance of ubiquitination and deubiquitination. E3 ligases and USP7 target DNA-bound, transcriptionally active NF-κB and do not control the upstream activation of NF-ĸB. As such, the ubiquitination of NF-ĸB represents an unexploited avenue for the therapeutic control of inflammatory disease.

We previously demonstrated that the inhibition of USP7 leads to the inhibition of NF-ĸB directed transcription of pro-inflammatory cytokines such as *Il1b*, *Tnf*, and *Il6*, identifying USP7 as a potential therapeutic target in inflammatory disease (13). However, USP7 deubiquitinates a growing number of substrate proteins, many with important roles in the regulation of key cellular processes such as cell cycle, differentiation, apoptosis, DNA replication, and transcription (14, 15). Indeed, the importance of two USP7 substrates in cancer, p53 and MDM2, has led to significant efforts to develop USP7 inhibitors. The currently available small molecule inhibitors of USP7 target the catalytic activity of USP7 and thus inhibit the activity of USP7 against all its substrates including p53, MDM2, and NF-ĸB (16). Although the impact of USP7 inhibitors on both cell survival (NF-ĸB) and cell death (p53/MDM2) pathways is desirable in the context of cancer therapy, such broad activity is unlikely to be beneficial in an anti-inflammatory agent.

In this study, we map the regions of USP7 involved in the interaction with the p65 (RelA) subunit of NF-ĸB, using peptide array technology, molecular modeling, and mutagenesis approaches. We identify the ubiquitin-like (Ubl) domain 2 of USP7 as critical for the interaction and deubiquitination of p65 but dispensable for the interaction of USP7 with the USP7 substrates p53, DAXX, and EBNA1. The Ubl2 domain of USP7 is also required for interaction with the c-Rel subunit but not the RelB subunit of NF-ĸB. Of note, we find the Ubl2 domain of USP7 is not required for USP7-mediated deubiquitination of RelB. The findings described here suggest that targeting noncatalytic domains of USP7 may enable substrate-selective inhibition of USP7 activity. Toward this goal we further describe a p65-binding site in the Ubl2 domain of USP7 that may facilitate the selective inhibition of USP7-mediated deubiquitination of p65.

## Results

### USP7 inhibition selectively inhibits TLR-induced NF-ĸB target gene expression

**Figure 1.**
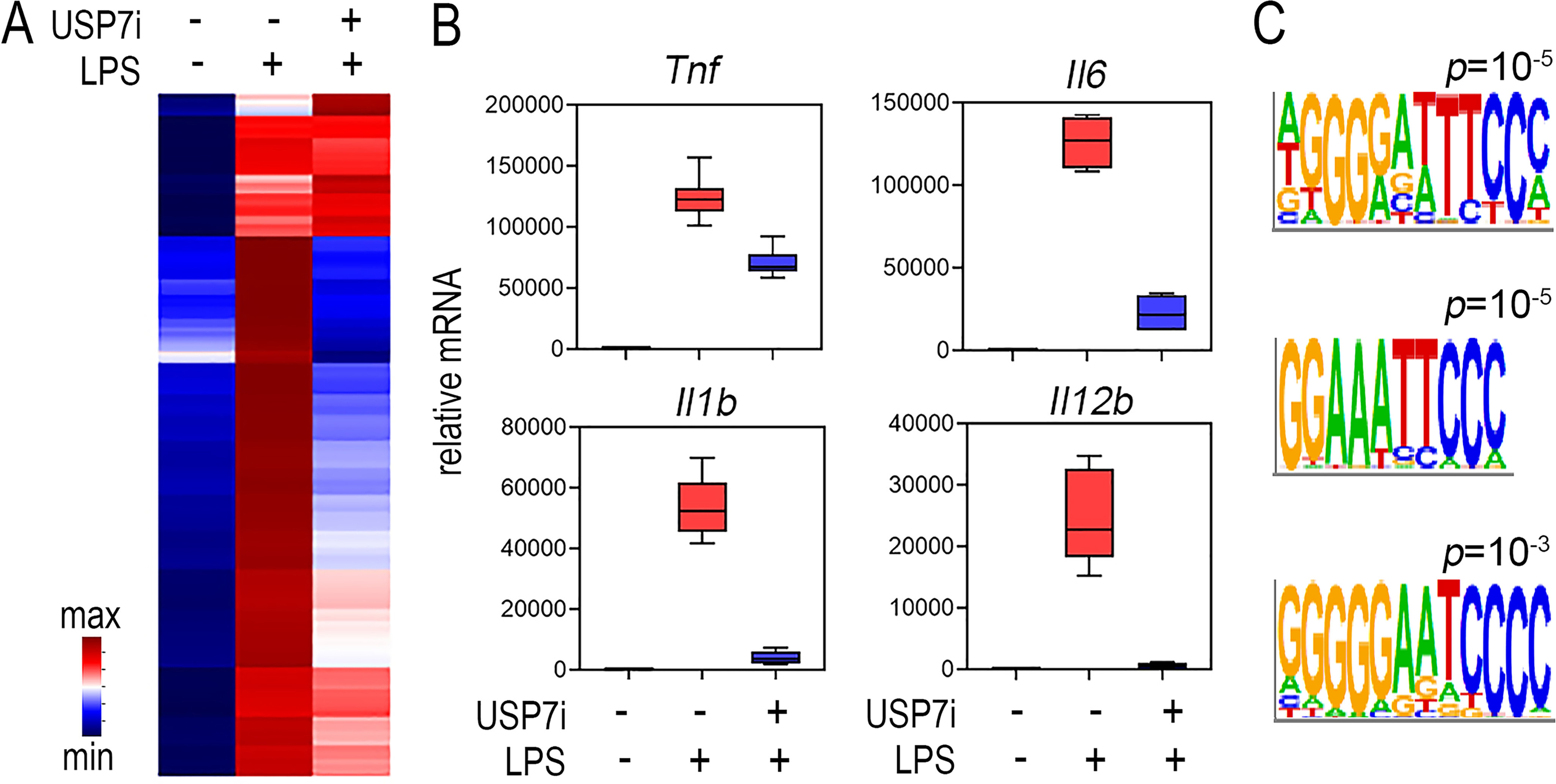
**USP7 inhibition selectively inhibits LPS-induced expression of NF-ĸB target genes.** Murine bone marrow–derived macrophages were stimulated with LPS (100 ng/ml) with or without 30 min of pretreatment with the USP7 inhibitor HBX14,108 (USP7i) (10 μm), and gene expression was analyzed by microarray. *A*, hierarchical clustering shows selective inhibition of LPS-induced gene expression by USP7 inhibitor treatment. The heat map displays differentially expressed genes (*p*.adj < 0.05) scaled as per *z* score. The values shown are the mean of duplicate samples analyzed. *B*, box and whisker plots of microarray data for *Tnf*, *Il6*, *Il1b*, and *Il12b*. *C*, the promoter regions of 1008 genes (transcriptional start site ± 2000 bp) inhibited by USP7 inhibitor treatment were analyzed for the occurrence of transcription factor–binding sites using HOMER. These genomic regions were significantly enriched in motifs corresponding to NF-ĸB–binding sites. Shown are sequence logos for the identified NF-ĸB motifs and their associated *p* values.

We have previously demonstrated that USP7 deubiquitinates NF-ĸB p65 to promote NF-ĸB transcriptional responses induced by tumor necrosis factor α and TLR ligands (13). Our earlier studies showed that inhibition of USP7 by the small molecule inhibitor HBX41,108 or siRNA knockdown blocked the expression of key inflammatory cytokines including *Tnf* and *Il6* (13). To further characterize the impact of USP7 inhibition on TLR-induced transcriptional responses, we performed a microarray-based transcriptomic analysis of murine bone marrow–derived macrophages (BMDMs) stimulated with the TLR4 ligand lipopolysaccharide (LPS) in the presence or absence of the USP7 inhibitor HBX41,108 (17). Hierarchical clustering analysis of LPS-induced genes demonstrated that the USP7 inhibitor reduced the expression of a significant number of TLR-inducible genes in macrophages (Fig. 1*A*), including the NF-ĸB target genes encoding inflammatory cytokines *Tnf*, *Il6*, *Il1b*, and *Il12b* (Fig. 1*B*). To further investigate the impact of USP7 inhibition on NF-ĸB–mediated TLR-induced transcriptional responses, we next analyzed the promoter sequences of genes inhibited by USP7 inhibitor treatment for the enrichment of transcription factor–binding sites. This analysis revealed a significant over-representation of NF-κB–binding sites in TLR-inducible genes inhibited by USP7 inhibitor treatment (Fig. 1*C*). These promoter regions were also enriched in binding sites for PU.1 and interferon response factors, transcription factors that cooperate with NF-ĸB to promote the expression of pro-inflammatory genes (Table S1). These data demonstrate that USP7 inhibition is an effective approach to block NF-ĸB–mediated inflammatory responses. However, the expanding list of identified USP7 substrates means that the inhibition of UPS7 catalytic activity will not achieve a selective effect on NF-ĸB activity. Instead, strategies that inhibit USP7 activity in a substrate-specific manner are required, which necessitates a detailed analysis of the recognition of p65 by USP7.

### The Ubl2 domain of USP7 is required for interaction with NF-ĸB p65

**Figure 2.**
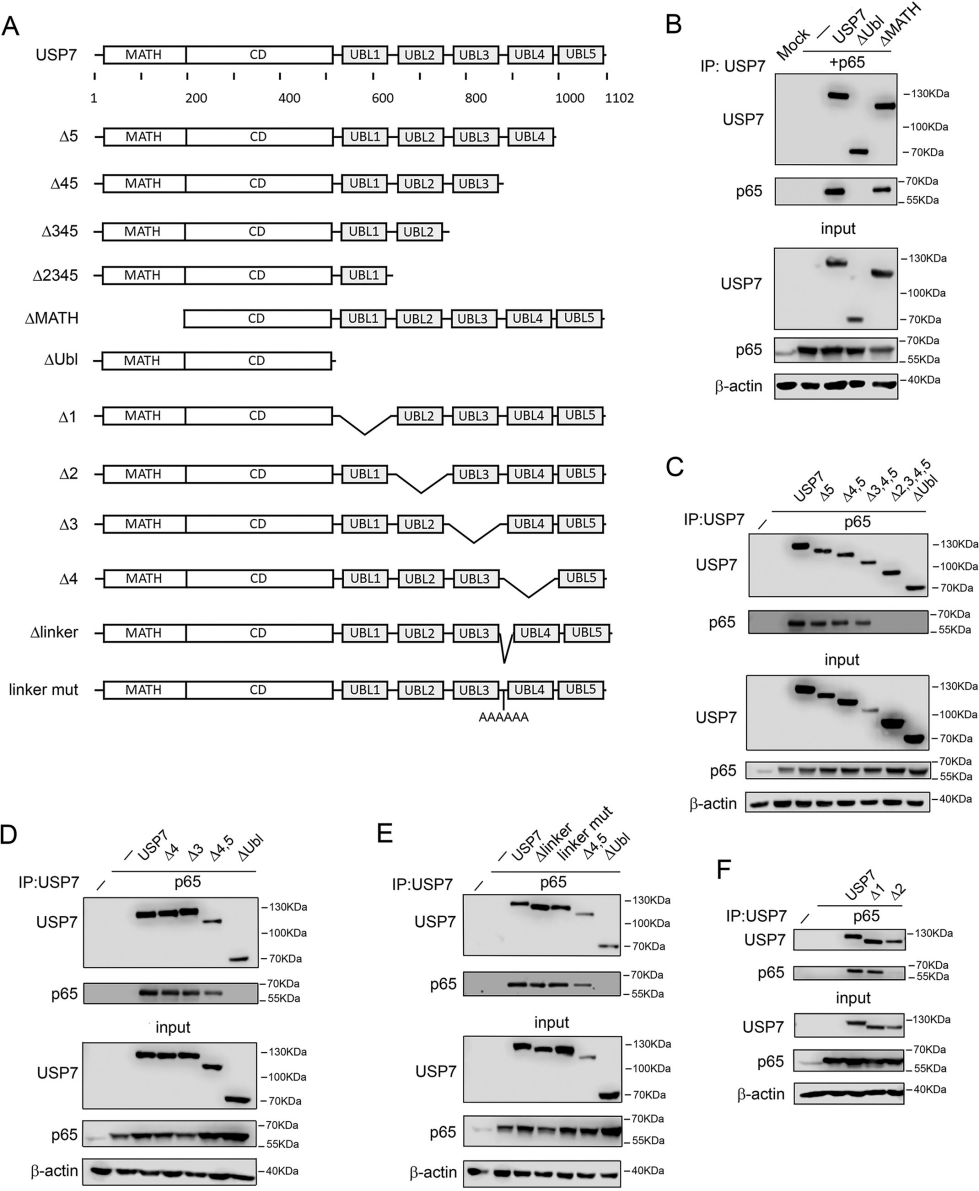
**The Ubl2 domain of USP7 is required for interaction with p65.** *A*, schematic representation of USP7 mutations used to test interaction with p65. USP7 contains an N-terminal MATH domain, a central catalytic domain (*CD*), and five C-terminal Ubl domains. *B–F*, HEK293T cells were transfected with p65 and the indicated FLAG-tagged USP7 plasmids. USP7 was immunoprecipitated (*IP*) from whole cell lysates using anti-FLAG antibody and analyzed by Western blotting (*WB*) with anti-p65 antibody. The expression of p65 and USP7 in lysates used for immunoprecipitation (*input*) was measured by Western blotting using anti-FLAG and anti-p65 antibodies. Western blotting of inputs with anti–β-actin was used to verify equal protein loading. The positions of molecular mass markers are indicated to the *right* of each Western blot. The data are representative of at least three independent experiments.

Our previous studies revealed that the C-terminal region of USP7, containing five Ubl domains, is essential for interaction with p65, whereas the N-terminal MATH domain is dispensable (13) (Fig. 2*A*). To further define the C-terminal regions of USP7 that mediate interaction with p65, we next assessed the contribution of specific Ubl domains of USP7. To do this, we generated a series of USP7 mutants in which each of the five Ubl domains were deleted either individually or in combination (Fig. 2*B*). Immunoprecipitation assays using whole cell lysates from cells co-transfected with p65 revealed that the Ubl5, Ubl4, and Ubl3 domains of USP7 were dispensable for interaction with p65. Thus, the deletion of Ubl5, Ubl4, and Ubl3 either individually or together did not prevent the interaction of USP7 with p65 (Fig. 2, *C* and *D*). Deletion or mutation of the linker region between Ubl3 and Ubl4 also did not prevent interaction with p65 (Fig. 2*E*). Furthermore, the deletion of Ubl1 did not inhibit USP7 interaction with p65 (Fig. 2*F*). However, deletion of Ubl2, either alone or in combination with the deletion of Ubl3, Ubl4, and Ubl5, prevented the interaction of USP7 with p65 (Fig. 2, *C* and *F*). These data demonstrate that the Ubl2 domain of USP7 is essential for the interaction with p65.

### Selective requirement for Ubl2 in USP7 substrate interactions

**Figure 3.**
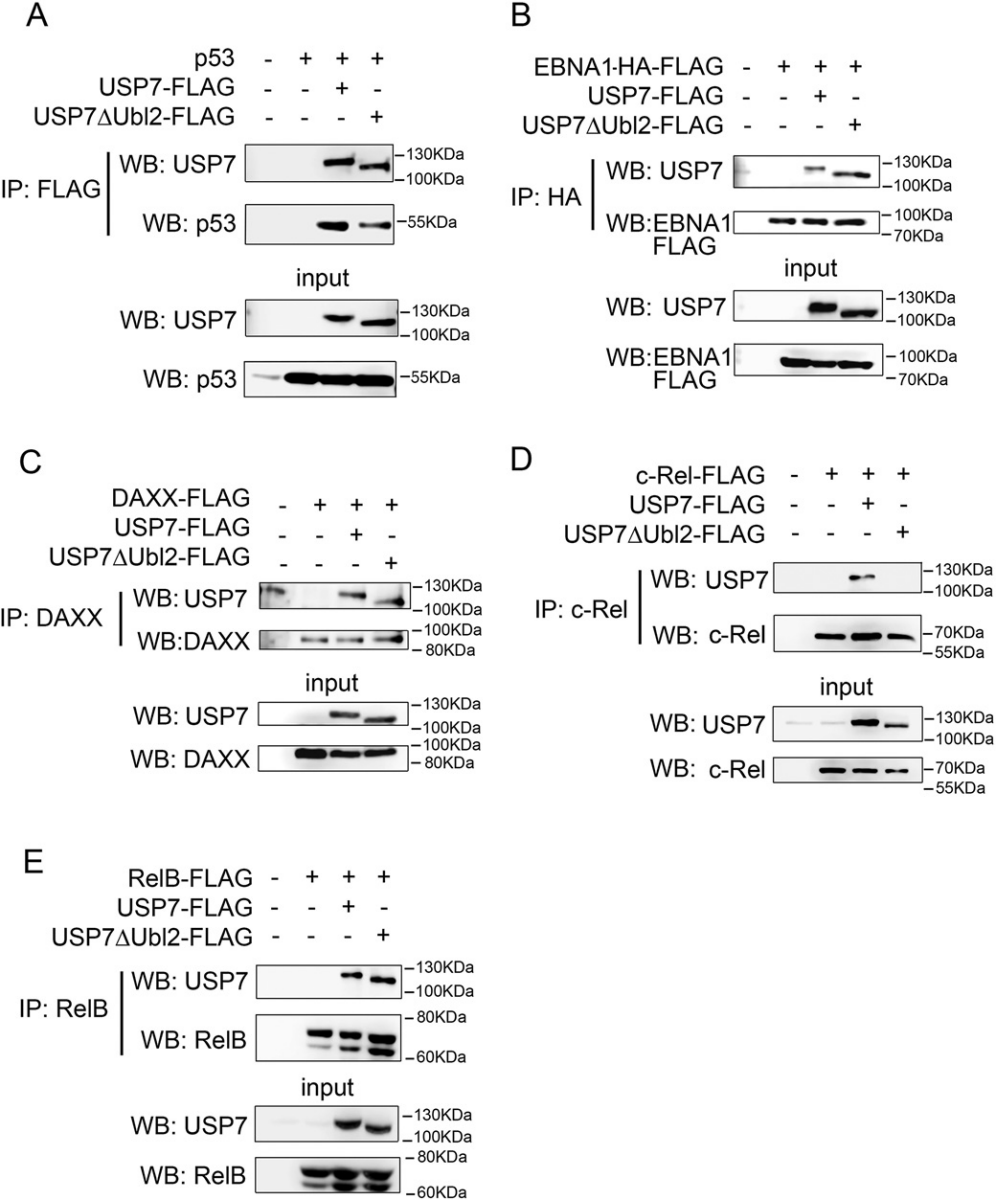
**The Ubl2 domain of USP7 is required for interaction with c-Rel but not for interaction with p53, EBNA1, DAXX, or RelB.** HEK293T cells were transfected with the indicated expression plasmids and whole cell lysates immunoprecipitated (*IP*) with anti-FLAG (*A*), anti-HA (*B*), anti-DAXX (*C*), anti-c-Rel (*D*), or anti-RelB (*E*). The expression of USP7-FLAG, USP7ΔUbl2-FLAG, p53, EBNA1-HA-FLAG, DAXX-FLAG, c-Rel-FLAG, and RelB-FLAG in whole cell lysates used for immunoprecipitations (*input*) was measured by Western blotting (*WB*) analysis with the indicated antibodies. The positions of molecular mass markers are indicated to the *right* of each Western blot. The data are representative of at least three independent experiments.

To determine the contribution of the Ubl2 domain to the interaction of USP7 with other substrates, we next assessed USP7 binding to selected substrates by immunoprecipitation assays using cells transfected with a mutant of USP7 lacking Ubl2 (USP7ΔUbl2). The results of these experiments demonstrated that the deletion of Ubl2 did not inhibit the interaction of USP7 with p53 or EBNA1 (Fig. 3, *A* and *B*). These findings support previous studies indicating that the N-terminal MATH domain of USP7 mediates interaction with p53 and EBNA1 (18, 19). We also tested the ability of USP7ΔUbl2 to interact with DAXX, a substrate for which the contribution of the C-terminal of USP7 to the interaction is unknown (20). Similar to p53 and EBNA1, immunoprecipitation experiments using cells co-transfected with DAXX and USP7ΔUbl2 revealed that the deletion of Ubl2 did not affect the interaction of USP7 and DAXX (Fig. 3*C*). In similar experiments, we also tested the requirement for Ubl2 in the interaction of USP7 with the NF-ĸB subunits c-Rel and RelB, which share significant sequence and structural homology with p65. This revealed that, similar to p65, the interaction of c-Rel with USP7 requires the Ubl2 domain of USP7 (Fig. 3*D*). Remarkably, however, and in contrast to c-Rel and p65, the deletion of Ubl2 did not inhibit the interaction of USP7 with RelB (Fig. 3*E*). These data demonstrate that the Ubl2 domain of USP7 is selectively required for the interaction of USP7 with its substrates, including among homologous members of the NF-ĸB family of transcription factors.

**Figure 4.**
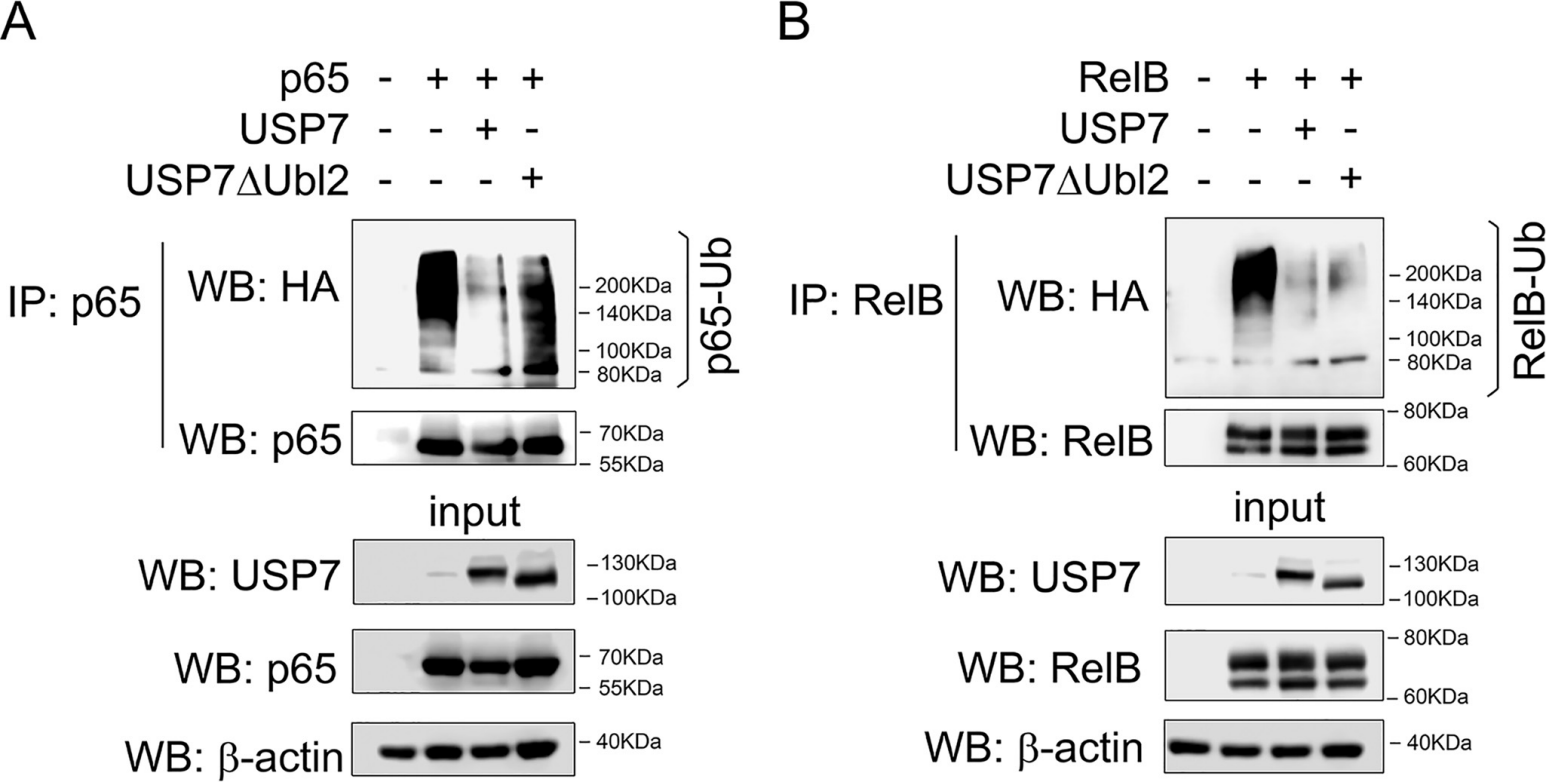
**The Ubl2 domain is required for USP7 deubiquitination of p65 but not RelB.** HEK293T cells were transfected with HA-tagged ubiquitin, USP7, USP7ΔUbl2, and p65 (*A*) or RelB (*B*). Denatured whole cell lysates were immunoprecipitated with anti-p65 (*A*) or anti-RelB (*B*) and analyzed by Western blotting (*WB*) with anti-HA antibody and anti-p65 (*A*) or anti-RelB (*B*) antibody. The expression of transfected USP7, USP7ΔUbl2, p65, and RelB in lysates used for immunoprecipitations (*IP*, *input*) was measured by Western blotting using antibodies against USP7, p65, and RelB. The positions of molecular mass markers are indicated to the *right* of each Western blot. The data are representative of at least three independent experiments.

We previously demonstrated that p65 is a direct substrate of USP7 and that interaction of p65 with USP7 is required for USP7-mediated deubiquitination of p65 (13). We next tested the ability of USP7ΔUbl2 to deubiquitinate p65 and RelB in an in-cell ubiquitination assay. The cells were transfected with HA-tagged ubiquitin, WT USP7 or USP7ΔUbl2, and RelB or p65. Following denaturing lysis, RelB or p65 were immunoprecipitated and immunoblotted with anti-HA antibody. This analysis revealed that WT USP7 but not USP7ΔUBL2 deubiquitinated p65 (Fig. 4*A*), consistent with the inability of USP7ΔUbl2 to interact with p65 (Fig. 3). In contrast, both WT USP7 and USP7ΔUbl2 were able to deubiquitinate RelB, demonstrating that Ubl2 is dispensable for USP7 interaction with and deubiquitination of RelB (Fig. 4*B*).

### Analysis of USP7 and NF-ĸB p65 binding by peptide array

**Figure 5.**
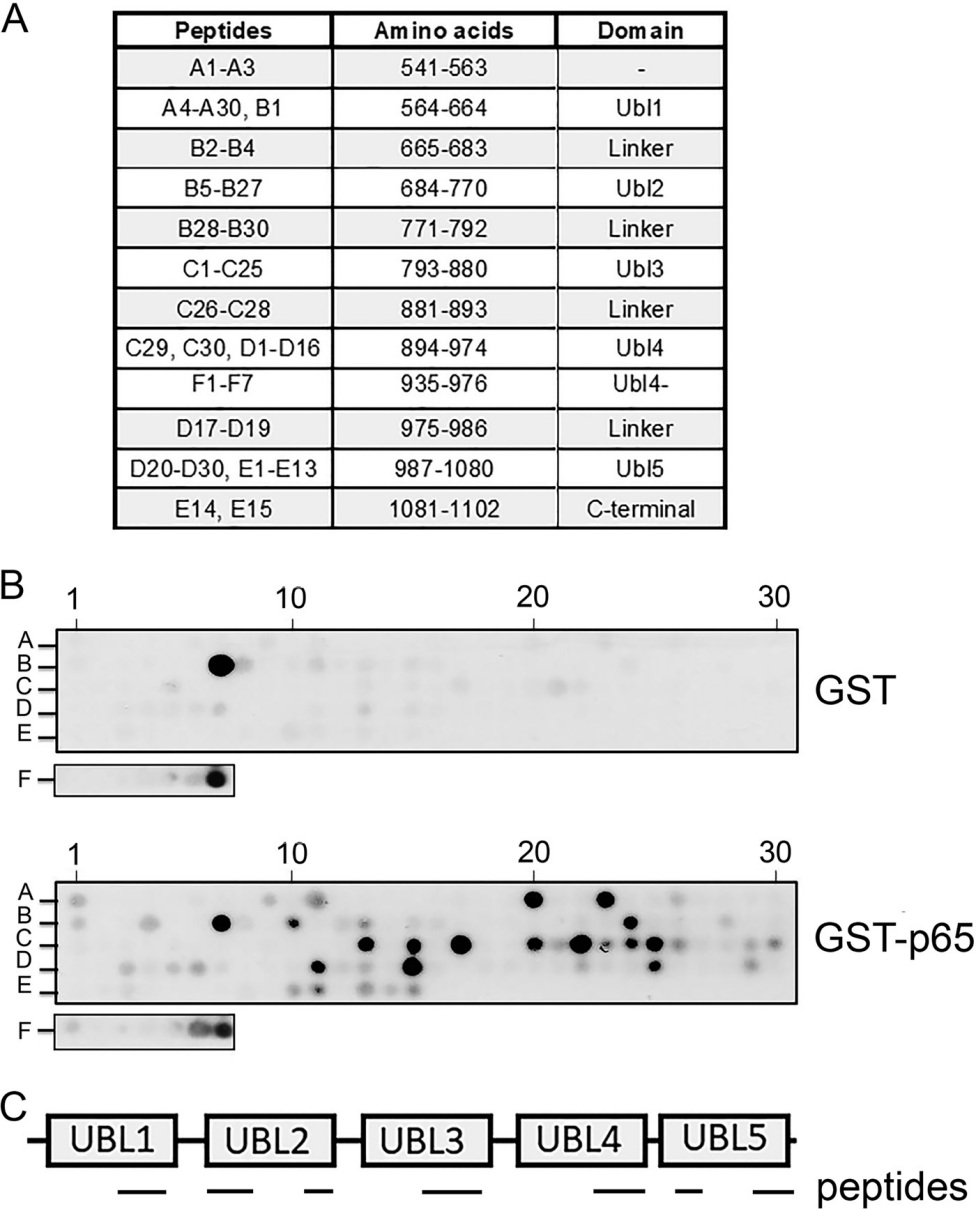
**Peptide array analysis identifies distinct regions of USP7 Ubl domains with potential for interacting with p65.** *A*, peptide arrays of immobilized overlapping 18-mer peptides, each shifted to the C terminus by 4 amino acids, encompassing the C-terminal region of USP7 were generated. Peptide identifiers and the corresponding amino acids and domain location in USP7 are shown. *B*, arrays were probed with recombinant GST or GST-p65 and detected by immunoblotting with anti-GST antibody. Positive GST-p65 binding to USP7 peptides is indicated by *black spots*. The data shown are representative of duplicate arrays. Peptide identifiers shown in *A* are indicated. *C*, schematic of the C-terminal regions of USP7 showing the location of peptides that interact with p65 identified in the peptide array.

Following the identification of the Ubl2 domain as essential for the interaction of USP7 with p65, we next sought to further define the specific regions of USP7 that mediate interaction with p65. To do this we employed a peptide array–based approach in which an immobilized peptide library representing the C-terminal region of USP7 was probed with purified recombinant GSH *S*-transferase (GST)–p65 protein. Specifically, a library of overlapping peptides 18 amino acids in length representing the C-terminal region of USP7 (amino acids 541–1102) was synthetic peptide arrays on membrane support (SPOT)–synthesized on nitrocellulose membranes to generate USP7 C-terminal arrays (Fig. 5*A*). The peptide arrays were overlaid with purified recombinant GST or GST-p65 protein, and interaction with specific peptides was detected using anti-GST antibody as previously described (21). This analysis revealed a distinct set of peptides that interacted strongly with GST-p65 but not GST. These peptides corresponded to sequences in each of the Ubl1, Ubl2, Ubl3, Ubl4, and Ubl5 domains of USP7, indicating potentially broad contacts with p65 across the C-terminal of USP7 (Fig. 5, *B* and *C*).

**Figure 6.**
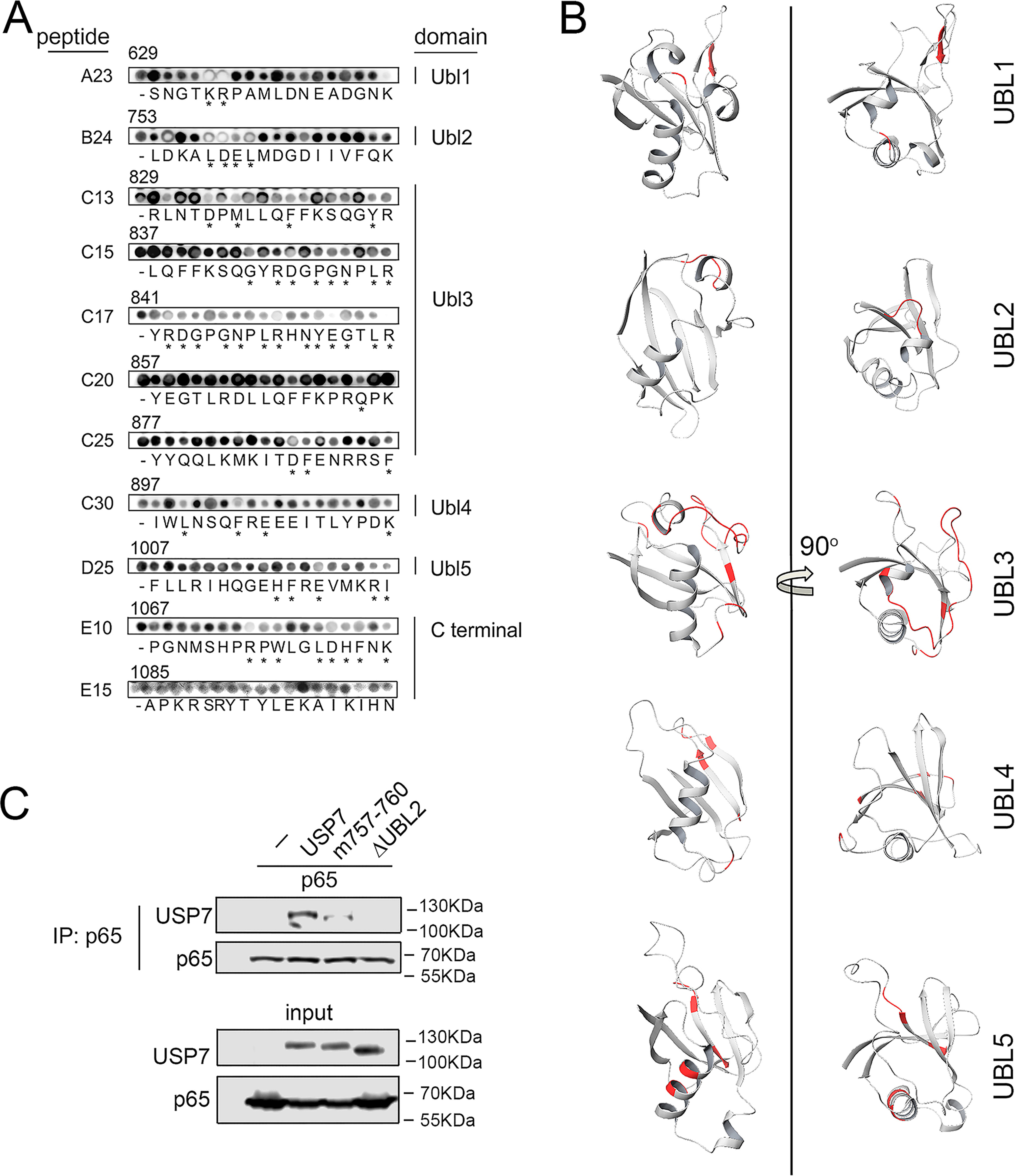
**Identification of amino acids important for USP7 interaction with p65.** *A*, the 18 amino acids of USP7-derived peptides of interest were sequentially substituted with alanine, probed with GST-p65, and detected by immunoblotting with anti-GST antibody. The peptides are labeled according to Fig. 5. p65 binding was quantified and calculated as a percentage binding of the parent unsubstituted peptide (-) on the same array. Alanine substitutions that resulted in less than 60% binding are indicated by *asterisks*. The corresponding USP7 amino acid number for first residue of each peptide is shown *above* each individual substitution peptide series. *B*, the structure of each Ubl domain with amino acids identified by alanine-scanning experiments indicated in *red*. The images were generated using Protein Data Bank structure 2YLM. *C*, HEK293T cells were transfected with p65 and FLAG-tagged USP7, USP7 mutated at residues 757–760, and USP7 lacking the Ubl2 domain (USP7ΔUbl2). p65 was immunoprecipitated (*IP*) from whole cell lysates using anti-p65 antibody and analyzed by Western blotting (*WB*) with anti-FLAG antibody. The expression of p65 and USP7 in lysates used for immunoprecipitation (*input*) was measured by Western blotting using anti-FLAG and anti-p65 antibodies. The positions of molecular mass markers are indicated to the *right* of each Western blot.

To identify specific amino acids within the identified regions of USP7 that may be important for interaction with p65, we next generated alanine-scanning arrays of selected peptides representing each Ubl domain and the C-terminal peptide region (Fig. 5*A*). The peptides in these arrays were modified such that successive amino acids were individually substituted with alanine and differed from the parent peptide by one amino acid. As before, peptide arrays were incubated with GST-p65, and binding was calculated with respect to the unmodified parent peptide contained on the same array. Substitutions with less than 60% binding of the parent peptide were considered as important amino acids for mediating the interaction of the peptide with p65. These data identified individual amino acids in each peptide that were required for interaction with p65. Of note, the individual substitution of four consecutive amino acids (LDEL) in a peptide representing amino acids 753–770 (^753^LDKALDELMDGDIIVFQK^770^) within the Ubl2 domain of USP7 blocked interaction with p65 (Fig. 6*A*). This identified the ^757^LDEL^760^ motif of USP7 as a potential binding site for p65. In addition, the alanine substitution of individual amino acids in peptides representing regions of USP7 in the other Ubl domains of the C-terminal protein region were also found to inhibit interaction with p65 (Fig. 6, *A* and *B*). This included a large number of amino acids in peptides corresponding to the Ubl3 domain, as well as the C-terminal peptide region. Although the data from experiments using USP7 mutants lacking individual Ubl domains showed that these domains are not essential for the interaction of USP7 with p65 (Fig. 3), it is possible that potential sites of interaction in these domains may also contribute to the interaction with p65, perhaps through stabilization of already bound p65.

### Lys^757^, Asp^758^, Glu^759^, and Lys^760^ mediate interaction of USP7 with p65

The alanine-scanning peptide array analysis identified USP7 amino acids Lys^757^, Asp^758^, Glu^759^, and Lys^760^ (^757^LDEL^760^) as residues that may mediate interaction of USP7 with p65. To investigate this we mutated these amino acids in USP7 and assessed the binding to p65 using immunoprecipitation assays. The cells were co-transfected with p65 and WT USP7 or a L757A, D758R, E759R, and L760A USP7 mutant, prior to immunoprecipitation using anti-p65 antibody and subsequent analysis by immunoblotting. The results of these experiments revealed that the mutation of Lys^757^, Asp^758^, Glu^759^, and Lys^760^ of USP7 significantly reduced interaction with p65 (Fig. 6*A*). Thus, these data identify a p65-binding site involving residues 757–760 of USP7 that mediates the selective binding to p65.

## Discussion

The importance of NF-ĸB in the regulation of pro-inflammatory gene expression has established it as a therapeutic target of significant potential (22). Previous strategies to inhibit NF-ĸB function focused largely on the development of inhibitors of the IKK kinases, key activators of the NF-ĸB pathway. Although the development of selective inhibitors of the IKK kinases was successful, their therapeutic use was precluded because of severe toxicity in early trials, possibly arising from the NF-ĸB–independent functions of the IKK kinases (23). Thus, the therapeutic potential of the NF-ĸB pathway remains untapped, and alternative approaches beyond inhibiting the IKK kinases are required.

The ubiquitin-triggered proteasomal degradation of the NF-ĸB subunits is an important mechanism for the termination of NF-ĸB transcriptional activity (13). The level of NF-ĸB–directed pro-inflammatory gene transcription is dictated by the balance between the action of NF-ĸB E3 ligases and the NF-ĸB deubiquitinase USP7. The inhibition of USP7 leads to enhanced ubiquitination and proteasomal degradation of NF-ĸB p65, resulting in the inhibition of pro-inflammatory gene expression (13). Here, we have performed the first transcriptomic analysis of the consequences of USP7 inhibition in macrophages stimulated with the TLR4 ligand LPS. This analysis reveals a significant and selective inhibition of NF-ĸB target gene expression following TLR4 activation and strongly supports the inhibition of USP7 deubiquitination of NF-ĸB as a potential therapeutic strategy.

Although the importance of USP7 in promoting NF-ĸB–mediated inflammatory responses is clear (13), targeting USP7 catalytic activity is unlikely to succeed where IKK inhibitors have failed. This is because, like the IKK kinases, USP7 has numerous substrates with important roles in a broad range of cellular processes, including cell cycle, DNA damage repair, and cell death. Instead, the therapeutic exploitation of USP7 control of NF-ĸB will require a substrate-selective approach. The data in this study represent a first step toward this goal. A p65 mutant that does not bind USP7 is hyperubiquitinated, has a shorter *t*_1/2_, and has significantly reduced transcriptional activity (13). Thus, interfering with USP7 and p65 interaction can achieve a substrate-selective inhibition of UPS7 mediated activity to inhibit the expression of pro-inflammatory genes. Our previous work identified the C-terminal domain of USP7 as a critical region required for interaction with p65 (13). In this study, we have further defined the Ubl2 domain of USP7 as essential for the interaction of USP7 with p65. In addition, we find that the Ubl2 domain is also essential for the interaction of USP7 with the c-Rel subunit of NF-ĸB but not the closely related RelB subunit. The finding that different domains of USP7 are important for the recognition of related substrates such as the NF-ĸB subunits is unexpected and suggests that substrate recognition by USP7 likely occurs through diverse interfaces, even among structurally related proteins. Of note, our data also show that the Ubl2 domain of USP7 is not required for the deubiquitination of RelB by USP7, suggesting that the Ubl2 domain functions primarily in substrate recognition rather than contributing to catalytic activity. Previous studies have shown the Ubl2 domain to be important for USP7 interaction with ICP0, UHRF1, and DHX40 (18, 24, 25), whereas the Ubl1 domain is essential for USP7 interaction with RNF168 (26). A number of USP7 substrates do not require the C-terminal of USP7 for interaction, including MDM2, p53, and EBNA1 (18). Indeed, a recent proteomics-based screen to identify USP7-interacting proteins revealed that most interactions analyzed occurred through the N-terminal MATH domain of USP7 (25). Together with this study, these data indicate that targeting USP7–substrate interactions may allow for the selective inhibition of USP7 activity.

Within the Ubl2 domain, we have identified the amino acids ^757^LDEL^760^ as important contributors to the interaction with p65. These residues may form part of a p65-binding pocket within the Ubl2 of USP7 that is important for the interaction with p65. This binding site is adjacent to, but distinct from, a binding site for UHRF1, IPC0, and GMPS formed by amino acids ^761^MDGD^764^. These studies, together with the data presented here, suggest that targeting the p65-specific binding site in USP7 may allow for the inhibition of p65 deubiquitination, leading to increased proteasomal degradation and reduced transcriptional activity, without broadly affecting the activity of USP7 toward all substrates. Similar approaches to mapping other substrate interaction sites of USP7 may lead to the identification of additional binding pockets that may be selectively targeted to achieve substrate-selective inhibition of USP7-mediated deubiquitination. Such an understanding of USP7 substrate interaction may facilitate the selective targeting of difficult to drug proteins such as NF-ĸB and other transcription factors.

## Experimental procedures

### Cell culture, plasmids, and transfection

Human embryonic kidney 293T (HEK293T) cells were cultured in high-glucose Dulbecco's modified Eagle's medium (DMEM) containing 10% heat-inactivated fetal bovine serum, 2 mm glutamine, and 100 units/ml penicillin/streptomycin. Murine BMDMs were generated as previously described (5). Briefly, bone marrow was isolated from age- and sex-matched C57BL6/J mice at 8–12 weeks old and cultured in bacterial Petri dishes with DMEM containing 10% fetal bovine serum, 2 mm glutamine, and 100 units/ml penicillin/streptomycin supplemented with 30% L929 conditioned medium for 7 days, with medium replacement after 4 days. On day 7, BMDMs were harvested in PBS solution supplemented with 5 mm EDTA and replated into tissue culture–treated dishes in DMEM without L929 conditioned medium. They were rested overnight, and the experiments were performed on day 8. The cells were maintained at 37 °C in a humidified environment with 5% CO_2_. These experiments were carried out in accordance with the recommendations of the Code of Practice for the Humane Killing of Animals under Schedule 1 to the United Kingdom Animals (Scientific Procedures) Act 1986, where excess tissue was used for isolation of bone marrow cells. Transfections were performed using Turbofect transfection reagent according to the manufacturer's instructions (Fermentas). Mammalian expression vectors for USP7, p65, c-Rel, RelB, and DAXX were generated following PCR amplification and ligation of cDNAs into the pcDNA-FLAG or pRK5-FLAG vectors. MSCV-N EBNA1 was a gift from Karl Munger (Addgene plasmid 37954). pCMV-p53 plasmid was purchased from Clontech. pET-42a-GST-p65 plasmid was generated by gene synthesis (GenScript). Site-directed mutagenesis was performed using the Q5 site-directed mutagenesis kit according to the manufacturer's instructions (New England Biolabs).

### Gene expression analysis

Total RNA was isolated using the RNeasy mini kit (Qiagen) with DNase treatment. RNA quantification and purity were measured using the Nanodrop ND1000 spectrophotometer (Thermo Scientific), with samples submitted for microarray profiling (Beckman Coulter Genomics, Morrisville, NC). Briefly, 200 ng of total RNA was fluorescently labeled with Cy3 nucleotides. Labeled RNA (cRNA) was hybridized to Agilent mouse 8X60K mouse microarrays (Agilent-028005). Hybridized arrays were washed and scanned, with data extrapolated for downstream bioinformatics analysis. Microarray gene-expression profiling was performed in duplicate. The data were normalized using Limma (3.38.3), including quantile normalization (27). A linear model was fitted to identify differentially expressed probes using an adjusted *p* value (Benjamini and Hochberg) of 0.05. The data are available at the NCBI Gene Expression Omnibus (GSE149478). Transcription factor–binding site analysis was performed using HOMER (RRID:SCR_010881).

### Bacterial protein expression and purification

For purification of recombinant GST-p65, *Escherichia coli* BL21 DE3 (Agilent Technologies) were transformed with pET-42a-GST-p65. The cultures were induced with 0.5 mm isopropyl β-d-1-thiogalactopyranoside and incubated for 3 h with shaking (150 rpm) at 37 °C. The bacteria were resuspended in buffer containing 50 mm Tris (pH 8.5), 150 mm NaCl, and 10 mm DTT, disrupted by sonication, and centrifuged to remove debris. GST-p65 was purified using GSH–agarose (Sigma) and eluted in buffer containing 50 mm Tris (pH 8), 10 mm DTT, and 10 mm reduced GSH (Promega).

### SPOT synthesis of peptides and overlay analysis

Peptide libraries of USP7 were generated by automatic SPOT synthesis as previously described (21). The interaction of GST and GST-p65 proteins with the peptide array was investigated by overlaying the peptide array membrane with 10 μg/ml of each protein overnight at 4 °C. Bound GST-p65 was measured by immunoblotting with anti-GST antibody (Sigma) and anti-rabbit horseradish peroxidase–conjugated secondary antibody (GE Healthcare) prior to enhanced chemiluminescence (ECL) detection. Specific alanine-scanning arrays were generated using the same procedure, and bound protein was detected and quantified using a digital chemiluminescence scanner (LiCor).

### Western blotting analysis and immunoprecipitation

For Western blotting analysis, whole cell lysates were prepared from cells suspended in radioimmune precipitation assay (RIPA) buffer containing 50 mm Tris-HCl (pH 7.4), 0.1–1% Igepal, 0.25% deoxycholate, 150 mm NaCl, 1 mm EDTA, 1 mm phenylmethylsulfonyl fluoride, 1 mm NaF, 1 mm Na_3_VO_4_, 2 µg/ml aprotinin, 1 µg/ml pepstatin, and 1 µg/ml leupeptin. Lysates were resolved on SDS-PAGE gels and transferred to nitrocellulose, and membranes were immunoblotted with specific antibodies. Anti-p65 was obtained from Santa Cruz Biotechnologies (sc-80089 and sc-372) and Bethyl Laboratories (A301-824A); anti-USP7 was obtained from Bethyl Laboratories (A300-033A); and Santa Cruz Biotechnologies provided anti-p53 (sc-6243), anti-HA (sc-805), anti-c-Rel (sc-71), anti-DAXX (sc-7152), and anti-RelB (sc-226) antibodies. Anti-FLAG (F1804), anti-GST (G7781), and anti–β-actin (SAB 1305567) antibodies were supplied by Sigma. For immunoprecipitation, equal amounts of whole cell extracts were precleared for 30 min at 4 °C with protein G–agarose beads (Millipore) and immunoprecipitated with specific antibody overnight at 4 °C. The beads were washed three times in RIPA buffer and eluted by boiling in 2× sample buffer (100 mm Tris, pH 6.8, 20% glycerol, 4% SDS, 0.2% bromphenol blue, 200 mm β-mercaptoethanol). Equal volumes of eluted immunoprecipitates were analyzed by Western blotting. Ubiquitination assays were carried out as previously described (5). Briefly, the cells were incubated with 10 mm *N*-ethylmaleimide for 30 s and washed in PBS supplemented with 10 mm *N*-ethylmaleimide. The cells were lysed in 1% SDS, boiled for 5 min, and sonicated. Cleared lysates were diluted 1:10 in RIPA buffer supplemented with 20 mm *N*-ethylmaleimide. Immunoprecipitation was performed as above and analyzed by Western blotting to detect ubiquitinated protein.

## Data availability

The microarray data presented have all been deposited in the NCBI GEO database with the following accession number: GSE149478. All remaining data are contained within the article.

## Supplementary Material

Supporting Information
