# Supplementary material for: The deubiquitinase USP7 uses a distinct ubiquitin-like domain to deubiquitinate NF-ĸB subunits
Source: J Biol Chem. 2020 Jun 25;295(33):11754–63. doi: 10.1074/jbc.RA120.014113 (PMC7450122; doi:10.1074/jbc.RA120.014113)
Supplement: Supporting Information [file supp_295_33_11754__index.html]

The deubiquitinase USP7 uses a distinct ubiquitin-like domain to deubiquitinate NF-κB subunits — USP7 and NF-κB interaction — The deubiquitinase USP7 uses a distinct ubiquitin-like domain to deubiquitinate NF-ĸB subunits — USP7 and NF-ĸB interaction — Supporting Information 

# The deubiquitinase USP7 uses a distinct ubiquitin-like domain to deubiquitinate NF-ĸB subunits

## Supporting Information

- Supporting Information (to be published online) - Supplemental table 1
