# Supplementary material for: The deubiquitinase USP7 uses a distinct ubiquitin-like domain to deubiquitinate NF-ĸB subunits
Source: J Biol Chem. 2020 Jun 25;295(33):11754–63. doi: 10.1074/jbc.RA120.014113 (PMC7450122; doi:10.1074/jbc.RA120.014113)
Supplement: Supporting Information [file supp_RA120.014113_160483_2_supp_548477_qbyymn.pdf]

Supplemental Table 1. Transcription factor binding sites enriched in the promoter regions of 1008 LPS-induced genes inhibited by HBX 41,108 treatment in mouse bone marrow derived macrophages. Genomic regions 2000bp up- and down-stream of the transcription start site were analysed by HOMER. Shown are the sequence logos of over-represented motifs, the transcription factors, *P* values and the number of genes containing the identified motifs.

Motif	Name	P-value	# Genes with motif
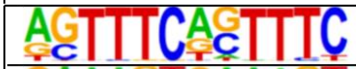	ISRE	1e-37	161
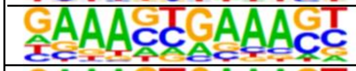	IRF2	1e-28	207
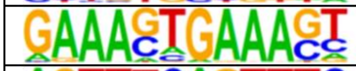	IRF1	1e-26	238
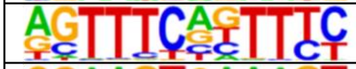	IRF3	1e-20	388
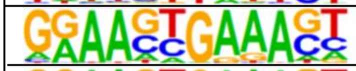	IRF8	1e-19	379
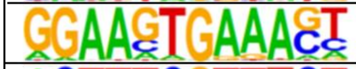	PU.1	1e-8	239
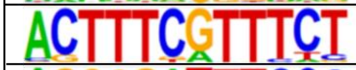	ISRE	1e-8	35
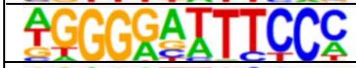	NFkB-p65	1e-5	427
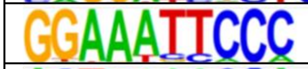	NFkB-p65-Rel	1e-5	88
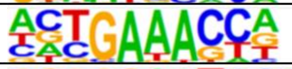	IRF4	1e-5	369
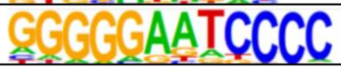	NFkB-p50,p52	1e-3	141
